# Can Routine Preoperative Endoscopy and Colonoscopy Be Meaningful for the Surgery and Postoperative Course of Patients with Advanced Ovarian Cancer? A Single-Centre Retrospective Cohort Study

**DOI:** 10.3390/diagnostics16193221

**Published:** 2026-10-05

**Authors:** Göktürk Han Doğan, Göksever Akpınar, Anıl Doğukan Tutal, Ceren Sağlam Purut, Ayşe Berin Acar, Mehmet Gökçü, Serhat Sarıkaya, Yunus Emre Purut

**Affiliations:** 1 Department of Gynecologic Oncology, İzmir Tepecik Training and Research Hospital, University of Health Sciences, 35020 Izmir, Türkiye; tutal1991@hotmail.com (A.D.T.); ayseberinacar@gmail.com (A.B.A.); purutemreyunus@hotmail.com (Y.E.P.); 2 Department of General Surgery, İzmir Tepecik Training and Research Hospital, University of Health Sciences, 35020 Izmir, Türkiye; gokseverakpinar@hotmail.com; 3 Department of Perinatology, İzmir Atatürk Training and Research Hospital, 35360 Izmir, Türkiye; drcerensaglam@yahoo.com; 4 Department of Gynecologic Oncology, Medical Point Hospital, İzmir University of Economics, 35575 Izmir, Türkiye; megokcu@yahoo.com; 5 Department of Obstetrics and Gynecology, İzmir Tepecik Training and Research Hospital, University of Health Sciences, 35020 Izmir, Türkiye; sarikayaserat@gmail.com

**Keywords:** ovarian neoplasms, cytoreduction surgical procedures, colonoscopy, endoscopy, gastrointestinal, colectomy, survival analysis

## Abstract

**Background/Objectives**: Bowel resection is often required for complete cytoreduction in advanced ovarian cancer, and some units perform upper gastrointestinal endoscopy and colonoscopy beforehand. We compared the endoscopic biopsy findings with the histopathology of the resected bowel and examined determinants of survival. **Methods**: Eighty-three consecutive women with FIGO stage III–IV serous ovarian cancer cytoreduced to no macroscopic residual disease between 2015 and 2021 were reviewed retrospectively; all had undergone both examinations and completed adjuvant carboplatin–paclitaxel. **Results**: Bowel resection was performed in 20 women (24.1%). Serosal involvement was reported in 15 specimens and no tumour in four; mucosal invasion was reported in none (95% CI 0–16.8%). Eighteen patients (21.7%) were biopsied endoscopically; no biopsy was malignant. Over a median follow-up of 72 months, 50 women (60.2%) developed recurrence (28 confirmed histologically and 22 diagnosed from imaging and CA-125; recurrence was ascertained retrospectively, which limits the interpretation of disease-free survival) and 44 (53.0%) died. Recurrence was no more frequent in biopsied than in non-biopsied patients (8/18 vs. 42/65, *p* = 0.174) or after resection (10/20 vs. 40/63, *p* = 0.306). Age showed an exploratory association with disease-free (HR 1.03/year, 95% CI 1.00–1.06) and overall survival (HR 1.04, 1.00–1.07). **Conclusions**: In this cohort, no preoperative endoscopic biopsy was malignant and no resected specimen was reported to show mucosal invasion, supporting a limited role for routine endoscopy in assessing bowel wall involvement; its diagnostic accuracy and its value for excluding a gastrointestinal primary could not be assessed.

## 1. Introduction

Ovarian cancer has the highest case-fatality rate among the gynaecological malignancies, with approximately 330,000 new diagnoses and more than 200,000 deaths worldwide in 2024 [1]. Most women present with advanced peritoneal disease [2], and complete cytoreduction to no macroscopic residual tumour, at primary or interval surgery, is the aim of treatment [3]; residual disease is a major prognostic determinant of survival [4]. Rectosigmoid involvement is one obstacle to that aim: resection of the affected segment often makes complete cytoreduction attainable but carries a risk of anastomotic leak [5].

The decision to resect is taken intraoperatively, on the basis of the appearance and mobility of the bowel. In a prospective series of 100 patients, tumour infiltrated the bowel wall in 73% of resected specimens, reaching the muscularis in 31% and the mucosa in 14% [6]; the prognostic weight of the depth of invasion remains uncertain [7,8]. Some units therefore perform upper gastrointestinal endoscopy and colonoscopy before cytoreductive surgery, to exclude a gastrointestinal primary and sometimes to inform the extent of resection. Recent series have found only limited value in predicting the need for bowel resection [9,10], but the endoscopic findings have not been compared with the depth of involvement in the resected specimen.

At our institution both examinations were performed in every woman scheduled for cytoreductive surgery, irrespective of symptoms. In a cohort confined to women in whom complete cytoreduction was achieved and adjuvant chemotherapy was completed, we asked whether routine preoperative endoscopy and colonoscopy are meaningful for the operation and the postoperative course. We compared the preoperative endoscopic biopsy findings with the histopathology of the bowel segments subsequently resected and, with residual disease held at zero, examined the determinants of recurrence and death.

## 2. Materials and Methods

### 2.1. Study Design and Patients

**Figure 1:**
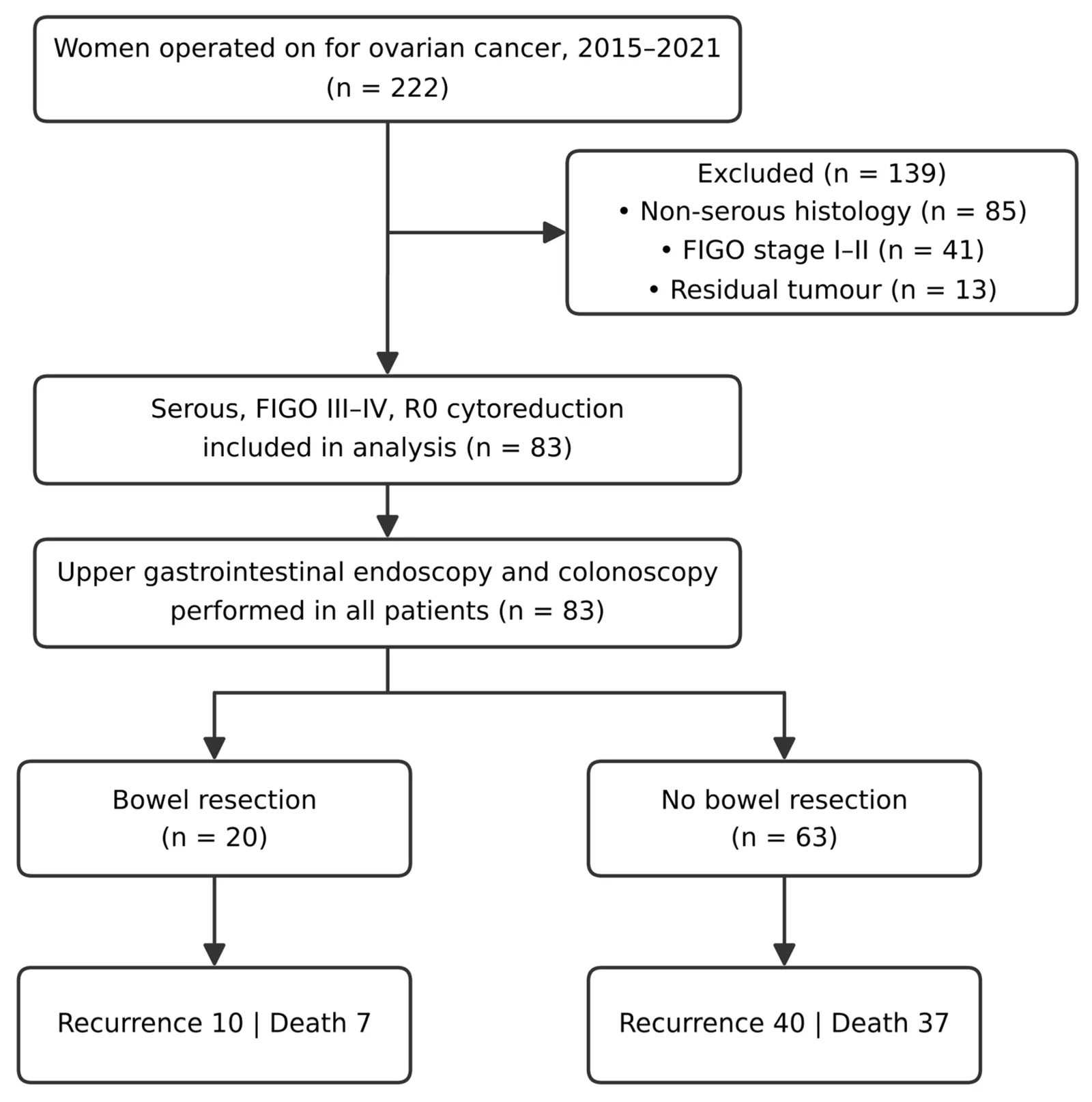
Flow of patients through the study.

This single-centre retrospective cohort study comprised 83 consecutive women who had undergone cytoreductive surgery for FIGO stage III–IV serous ovarian cancer between 2015 and 2021 and in whom no macroscopic residual tumour remained, as recorded by the operating surgeon. Of 222 women operated on for ovarian cancer in that period, 139 were excluded: 85 for non-serous histology (including women whose final pathology showed a gastrointestinal primary), 41 for stage I–II disease and 13 for residual tumour (Figure 1). All patients had received adjuvant carboplatin and paclitaxel and had completed six cycles, as verified against oncology records; the nine women who underwent interval cytoreduction had also received neoadjuvant chemotherapy. Dose intensity and treatment delays had not been recorded, and neither molecular profiling nor maintenance therapy was in routine use during the study period. All eligible patients were included without a sample size calculation. The study was approved by the Non-Interventional Clinical Research Ethics Committee of the University of Health Sciences, İzmir Tepecik Training and Research Hospital (approval number 2026/08-31, 9 September 2026), which waived the requirement for informed consent because of the retrospective design, and was conducted in accordance with the Declaration of Helsinki.

### 2.2. Preoperative Endoscopic Assessment

Institutional policy required upper gastrointestinal endoscopy and colonoscopy before cytoreductive surgery, and both examinations had been performed in all patients. Biopsy was taken at the endoscopist’s discretion when a mucosal abnormality was seen. The database recorded whether each examination had prompted a biopsy and the histological result of that biopsy; the endoscopic appearance itself was not recorded in a structured form. Endoscopic findings were not used to decide on bowel resection, which was determined intraoperatively by tumour involvement and the mobility of the segment.

### 2.3. Bowel Resection and Pathology

Resected specimens had been reported in narrative form by the institutional pathology department. Serosal and mucosal involvement were described in most reports, but involvement of the muscularis propria was not systematically recorded. The full text of each pathology report was reviewed, and the intraoperative indication for resection was abstracted from the operative note. For this study each report was assigned to one of three mutually exclusive categories: serosal involvement reported; tumour present with the layer not specified; and no tumour reported. Mucosal invasion was counted only where it was explicitly reported.

### 2.4. Variables and Outcomes

Age, body mass index, stage, preoperative CA-125, CEA and CA19-9, endoscopic biopsy results, bowel resection and its histopathology, hyperthermic intraperitoneal chemotherapy (HIPEC), primary versus interval cytoreduction, operating time, intraoperative blood loss and length of stay were recorded. The prognostic nutritional index (PNI) was calculated as 10 × serum albumin (g/dL) + 0.005 × total lymphocyte count (/mm^3^) [11] and was available in 81 patients. The peritoneal cancer index had been recorded only as predefined bands and was not analysed. All patients had undergone preoperative magnetic resonance imaging, and some had additionally undergone computed tomography or positron emission tomography–computed tomography; the imaging findings were not analysed in this study. BRCA and homologous recombination deficiency status were not recorded, as molecular profiling was not in routine use during the study period. Disease-free survival (DFS) was measured from diagnosis to first recurrence, and overall survival (OS) from diagnosis to death or the last visit. Follow-up data were last updated in September 2026; patients alive at that time were censored at their last documented visit. Surveillance comprised clinical examination and CA-125 every three months for two years and every six months thereafter, with annual computed tomography or magnetic resonance imaging, and positron emission tomography–computed tomography when relapse was suspected.

### 2.5. Ascertainment of Recurrence

Recurrence was first identified from the pathology records, which documented a histologically confirmed recurrence in 28 patients, 22 of whom subsequently died and 6 of whom were alive at last follow-up. Because recurrent ovarian cancer is not operated on or biopsied in every patient, recurrence was then ascertained clinically for the entire cohort: all follow-up imaging reports and serial CA-125 measurements were reviewed for each of the 83 patients. Recurrence was defined as new or progressive disease on cross-sectional imaging together with a rising CA-125; this single criterion was applied in the same way to every patient, and the date of its first documentation was taken as the date of recurrence. In this cohort, the two findings were concordant in every patient: each patient with a rising CA-125 had lesions consistent with recurrence on imaging, and each patient with such lesions had an elevated CA-125. On this basis, recurrence was identified in 50 patients: the 28 with histologically confirmed recurrence and a further 22, all of whom had died. Every death was therefore preceded by a documented recurrence, and no recurrence was identified among the remaining 33 patients, all of whom were alive. That histologically confirmed recurrences are fewer than all recurrences is expected, because most relapses of ovarian cancer are diagnosed clinically rather than surgically. Because the clinical ascertainment was performed retrospectively with vital status known, ascertainment bias cannot be fully excluded; all recurrence analyses are therefore also reported with the 28 histologically confirmed recurrences as a sensitivity analysis (Section 3.7), and overall survival, which does not depend on the ascertainment of recurrence, is reported alongside.

### 2.6. Statistical Analysis

Continuous variables are given as median (25th–75th percentile) and were compared with the Mann–Whitney U test; categorical variables were compared with Fisher’s exact test. Exact binomial 95% confidence intervals (CI) were calculated for proportions with no events. Survival was estimated by the Kaplan–Meier method and compared with the log-rank test, and median follow-up was estimated by the reverse Kaplan–Meier method. Cox regression with the Efron correction for ties was used, and proportional hazards were verified with scaled Schoenfeld residuals (lowest *p* = 0.195). Variables for the multivariable models were chosen on clinical grounds and univariable association, without stepwise selection: the DFS model comprised age, log_10_ CA-125, bowel resection and stage IV disease (12.5 events per variable), and the OS model comprised age, PNI, bowel resection and stage IV disease, fitted in the 81 patients with an available PNI (10.8 events per variable). Because the neoadjuvant period of interval cases falls within the interval measured from diagnosis, the survival analyses were repeated with the date of surgery as the time origin. A *p* value below 0.05 was considered significant, without adjustment for multiplicity. Analyses were performed in Python 3.13 (Python Software Foundation, Wilmington, DE, USA) with NumPy 2.5.3, SciPy 1.18.1 and statsmodels 0.15.0. Reporting follows the STROBE statement [12].

## 3. Results

### 3.1. Cohort

Median age was 60 years (51–66) and median CA-125 was 1157 U/mL (633–2760). Stage was IIIA in 5, IIIB in 14, IIIC in 47, IVA in 11 and IVB in 6 patients; 18 (21.7%) received HIPEC and 9 (10.8%) underwent interval cytoreduction. Median follow-up was 72 months. Women who developed recurrence were older and had higher CA-125 values, although neither difference was significant (Table 1).

### 3.2. Preoperative Endoscopic Findings

Upper gastrointestinal endoscopy and colonoscopy had been completed in all 83 women. Biopsy was taken from seven patients at colonoscopy and from 13 at upper endoscopy; two patients were biopsied at both examinations, so that 18 patients (21.7%) were biopsied in all (Table 2). The colonoscopic biopsies showed a polyp in two patients and non-specific inflammatory change in five; all upper endoscopic biopsies showed non-specific change. No biopsy showed malignancy, and no synchronous gastrointestinal primary was identified in this cohort. Among the 20 women who underwent bowel resection, four had been biopsied at colonoscopy and four at upper endoscopy; none of these biopsies was malignant.

### 3.3. Histopathology of the Resected Bowel

Twenty patients (24.1%) underwent bowel resection, which was rectosigmoid in 13 (Table 3). Serosal involvement was reported in 15 specimens, and in 10 of these the mucosa was explicitly described as uninvolved (in one, the submucosa as well). One specimen contained tumour with no layer specified, and four contained no reported tumour. Mucosal invasion was reported in none of the 20 specimens (95% CI 0–16.8%); restricted to the 14 specimens in which the mucosa was explicitly described as uninvolved or no tumour was reported, the upper bound was 23.2%. The indication recorded in the operative note was suspected serosal implants in 12 resections, rectosigmoid involvement requiring en bloc resection in three, a mass narrowing the bowel lumen in three, a mass infiltrating the bowel wall in one and multisegmental involvement in one. The five total or subtotal colectomies were performed for suspected serosal implants in three, multisegmental involvement in one and a mass narrowing the lumen in one. Three of the four specimens without reported tumour came from the resections performed for a mass narrowing the lumen, whose reports described inflammation or congestion only; the fourth came from a resection for suspected serosal implants. Table 4 lists the type of resection, the intraoperative indication and the histopathology for each of the 20 women; every specimen containing tumour from a total or subtotal colectomy or a left hemicolectomy showed serosal involvement. Preoperative colonoscopy had been performed in all 20 women; in the ten whose specimens contained no reported tumour, tumour with no layer specified, or serosal involvement without a description of the mucosa, it had shown no lesion prompting biopsy in eight, and the biopsies taken in the other two were not malignant.

### 3.4. Survival

**Figure 2:**
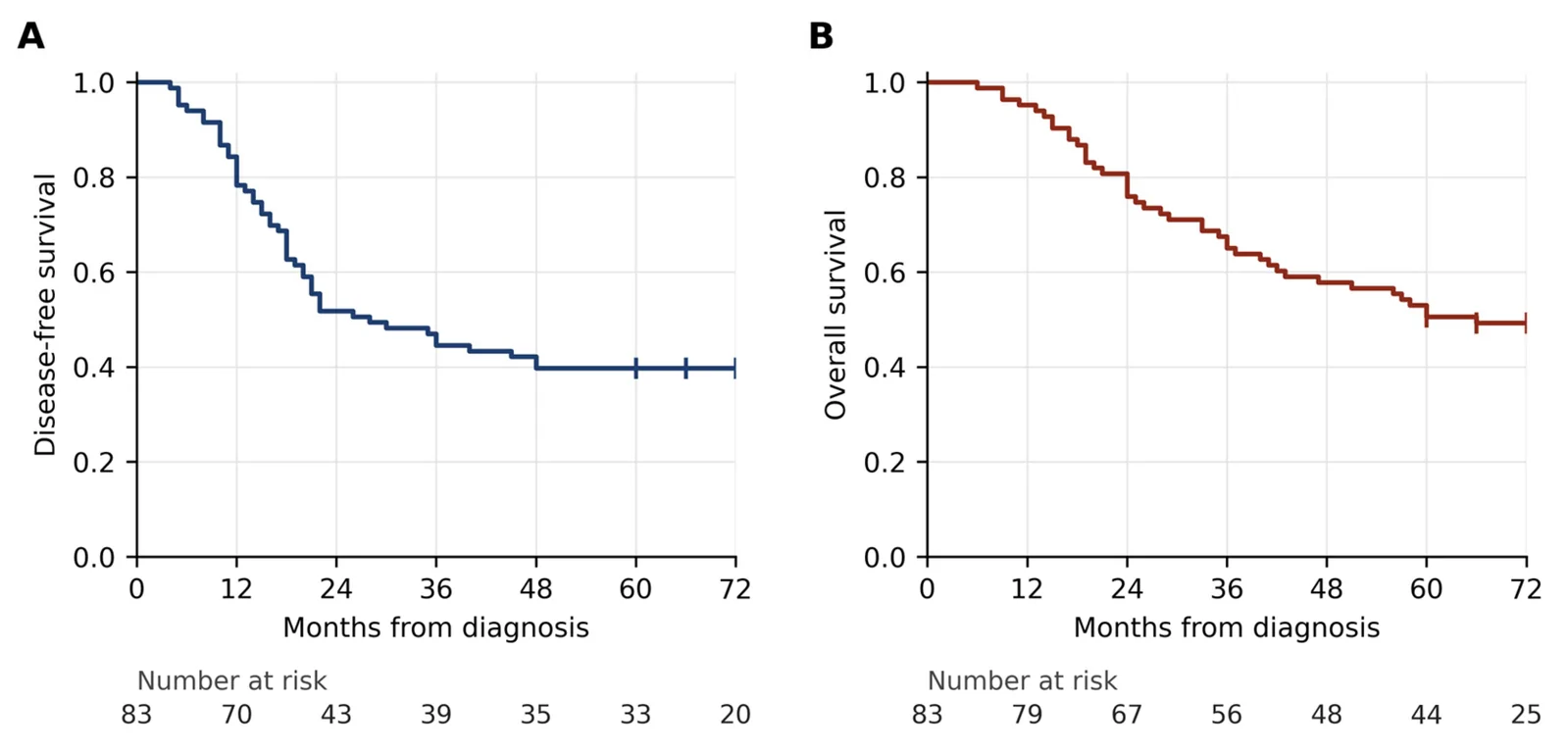
Kaplan–Meier estimates of (**A**) disease-free and (**B**) overall survival for the whole cohort (*n* = 83), truncated at 72 months. Vertical marks denote censored observations; numbers at risk are shown below each panel.

Fifty patients (60.2%) developed recurrence and 44 (53.0%) died. Median DFS was 28 months and median OS 66 months; DFS was 52%, 45% and 40% and OS 76%, 65% and 51% at two, three and five years (Figure 2). The 22 clinically diagnosed recurrences had been recorded closer to death than the histologically confirmed recurrences (median 6 [3–9] vs. 18 [15–26] months, *p* < 0.001).

### 3.5. Endoscopic Biopsy, Bowel Resection and HIPEC Subgroups

Recurrence and death were no more frequent in the 18 women who had been biopsied endoscopically than in the 65 who had not (recurrence 8/18, 44% vs. 42/65, 65%, *p* = 0.174; death 6/18, 33% vs. 38/65, 58%). Among the 20 women who underwent bowel resection, 10 (50%) developed recurrence and seven (35%) died, and neither median DFS nor median OS was reached; in the 63 women without resection, 40 (63%) developed recurrence and 37 (59%) died, with a median DFS of 22 months and a median OS of 57 months (log-rank *p* = 0.169 and *p* = 0.108). Recurrence was equally frequent with and without HIPEC (61% vs. 60%, *p* = 1.000).

### 3.6. Determinants of Survival

**Figure 3:**
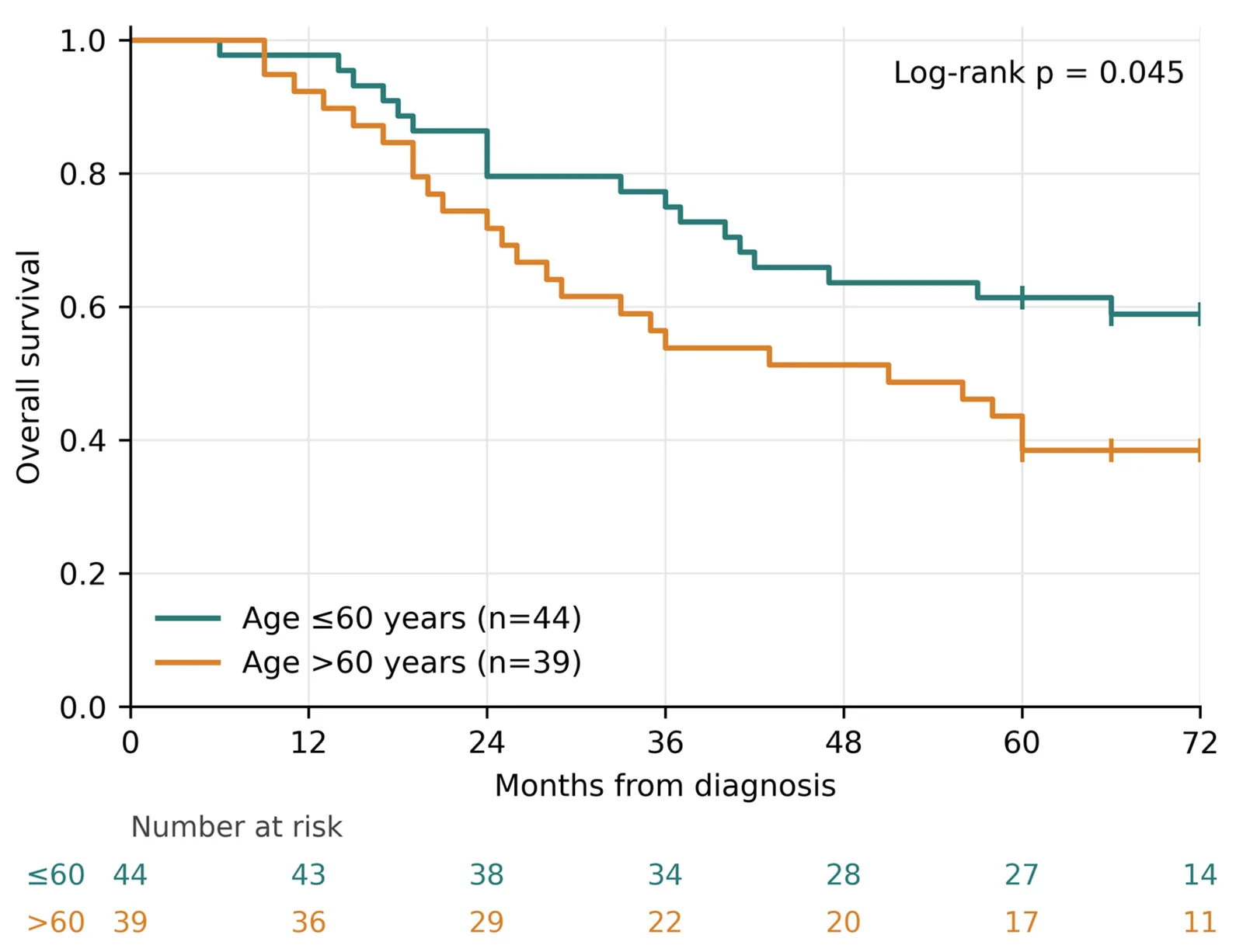
Overall survival by age group (median split at 60 years). The dichotomy is shown for illustration; inference rests on the Cox models with age as a continuous variable (Table 5).

On multivariable analysis, age was the only variable independently associated with DFS (HR 1.03 per year, 95% CI 1.00–1.06, *p* = 0.038) and OS (HR 1.04, 1.00–1.07, *p* = 0.033) (Table 5). Log_10_ CA-125 was associated with recurrence on univariable analysis (HR 1.87, 1.06–3.33) but not after adjustment (HR 1.69, 0.95–3.00); bowel resection, stage IV disease and PNI showed no independent association. The univariable hazard ratio below one for interval cytoreduction rested on nine patients with two events and was not interpreted. Overall survival was shorter in the 39 women older than the median of 60 years than in the 44 aged 60 years or less (log-rank *p* = 0.045; Figure 3), whereas the corresponding difference in DFS did not reach significance (*p* = 0.096).

### 3.7. Sensitivity Analyses

Table 6 sets out disease-free survival with all 50 recurrences and with the 28 histologically confirmed recurrences only, side by side. With histologically confirmed recurrences only, median DFS was not reached and DFS was 74%, 64% and 59% at two, three and five years, respectively. In this sensitivity analysis, the association between age and recurrence persisted (univariable HR 1.05, 1.01–1.10, *p* = 0.015; adjusted HR 1.05, 1.01–1.10, *p* = 0.016), whereas the association with CA-125 did not (HR 1.18, 0.56–2.47, *p* = 0.665); with 28 events the multivariable model had seven events per variable, and its estimates are correspondingly less precise. Neither endoscopic biopsy nor bowel resection was associated with recurrence under either definition. With the date of surgery as the time origin, the univariable hazard ratio for age was 1.03 for recurrence (*p* = 0.041) and 1.04 for death (*p* = 0.016). Overall survival does not depend on the definition of recurrence.

## 4. Discussion

In 20 bowel segments resected to achieve complete cytoreduction, mucosal invasion was reported in none, and no endoscopic biopsy in the cohort showed malignancy. In the largest prospective series the mucosa was the layer least often involved [6], so an examination that inspects it addresses the part of the bowel wall least likely to be affected.

Deeper involvement is nonetheless common in surgical series. Hertel et al. found the muscularis infiltrated in 31% and the mucosa in 14% of resected specimens [6], Mert et al. reported invasion of the muscularis, submucosa or mucosa in 38% [7], and in a series that included recurrent disease, implants reached the muscular or mucosal layer in three-quarters of cases [8]. Our series can be compared with those figures only for the mucosa, since the muscularis propria was not systematically described: no reported mucosal invasion (0%) against 14% in the prospective series of Hertel et al. [6]. In the specimens in which the mucosa was not described, preoperative colonoscopy had shown no malignant lesion; because mucosal involvement would usually be visible endoscopically, it is unlikely, although it cannot be excluded, that it was present in these women. Conversely, no tumour was reported in four of our 20 specimens (20%), close to the 27% of resected specimens without infiltration in the same series [6]; in both cohorts, therefore, a fifth to a quarter of the segments removed to achieve complete cytoreduction proved histologically free of tumour.

Liu et al. found that gastroscopy and colonoscopy had limited power to predict gastrointestinal resection and no advantage over imaging [9], and Endres et al. reported moderate sensitivity (67%) and specificity (74%) of colonoscopy for colorectal involvement, concluding that intraoperative assessment remained the primary determinant of colorectal intervention [10]. Our study adds the histopathology of the resected bowel. Both examinations were performed together in every patient, and in none of the 16 women whose resected bowel contained tumour had either examination shown any finding in favour of malignancy. Among the 20 women who needed resection, a colonoscopic abnormality prompting biopsy had been seen in four and an upper endoscopic abnormality in four, and none of those biopsies was malignant; the resected segments showed no reported mucosal invasion. Because ovarian cancer involves the bowel from the serosal surface, a negative mucosal biopsy is the expected finding and does not by itself show that endoscopy is uninformative; colonoscopy did identify polyps in two women. What this cohort supports is narrower: the endoscopic biopsies did not identify the bowel involvement that led to resection, so routine endoscopy appears to have limited value in assessing bowel wall involvement. Its other purpose, the exclusion of a gastrointestinal primary, could not be examined here, since women in whom such a primary was found were ineligible; in the series of Liu et al. these primaries almost always presented with indicative imaging or tumour marker abnormalities [9]. The exclusion of these women may itself explain why endoscopy appeared to contribute so little in this series, since those in whom it was most likely to be informative were not included by design. This finding is specific to the present series and remains open to debate.

An endoscopic abnormality prompting biopsy did not mark a worse prognosis: recurrence and death were, if anything, less frequent in the 18 biopsied women than in the rest of the cohort. Bowel resection was likewise not associated with poorer outcome, even though it was performed for bowel involvement apparent at operation; neither median DFS nor median OS was reached in that subgroup. Bowel resection is undertaken when it allows complete cytoreduction [5], so a relatively favourable outcome in this subgroup is not surprising; it most likely reflects this selection rather than any effect of resection itself, and no protective effect of bowel resection can be inferred. The HIPEC (*n* = 18) and interval cytoreduction (*n* = 9) subgroups were too small to be interpreted; the benefit of HIPEC, supported at interval cytoreduction [13] but not confirmed when primary surgery was included [14], could not be examined here.

With residual disease held at zero and chemotherapy completed in every patient, the only conventional variable that remained associated with both endpoints was age, and that association persisted when only histologically confirmed recurrences were counted and with surgery as the time origin, which suggests that it is not an artefact of the definition of recurrence. Age over 65 years has been independently associated with peritoneal recurrence after complete cytoreduction [15], whereas a propensity-matched study attributed the poorer outcome of older women to incomplete surgery and chemotherapy rather than to chronological age [16]; since every patient here completed chemotherapy, the latter explanation is less likely. Comorbidity and chemotherapy dose intensity, either of which could confound an association with age, were not measured, and the hazard ratios were modest (1.03–1.04 per year); the association is therefore exploratory and does not establish age as a prognostic factor. The CA-125 association did not persist when only histologically confirmed recurrences were counted and is not regarded as a finding. A low PNI has been associated with poorer prognosis in pooled analyses [17], but it did not reach significance here.

The strengths of this study are that both examinations were performed in every woman under institutional policy, irrespective of symptoms; that complete cytoreduction and completed adjuvant chemotherapy held residual disease and treatment completion constant; and that median follow-up was 72 months. It is, however, retrospective and single-centre. The endoscopic appearance was not recorded in structured form, so an abnormality could be identified only where it had prompted a biopsy; together with the exclusion of women in whom a gastrointestinal primary was found, this means that neither the diagnostic accuracy nor the yield of endoscopy could be estimated here. Muscularis propria involvement, preoperative imaging and postoperative complications were not analysed, and the peritoneal cancer index was available only in bands. Central review of the resected specimens was not feasible retrospectively, so the depth categories rest on the original narrative pathology reports. Twenty-two recurrences were diagnosed from imaging and CA-125 rather than histologically and were ascertained retrospectively with vital status known; this is usual in practice but is a less stringent definition than biopsy-proven relapse. The confidence intervals for bowel resection were wide, and the cohort predates maintenance therapy. The cohort is also highly selected: only women in whom complete cytoreduction was achieved and who completed six cycles of adjuvant chemotherapy were included, so women with residual disease, incomplete treatment or early death are not represented. The findings therefore apply to this favourable subgroup rather than to advanced ovarian cancer in general.

## 5. Conclusions

Mucosal invasion was not reported in any resected bowel specimen, and none of the preoperative endoscopic biopsies was malignant, including those taken from eight of the 20 women who required bowel resection. In this cohort, therefore, routine preoperative endoscopy appears to have limited value in assessing bowel wall involvement, although its diagnostic accuracy could not be estimated because the endoscopic appearance was not recorded in structured form. Whether endoscopy retains value for excluding a gastrointestinal primary could not be addressed here, because women in whom such a primary was found did not meet the inclusion criteria. Age was the only variable independently associated with both disease-free and overall survival, an association that persisted in sensitivity analyses but that remains an exploratory prognostic observation in a cohort of this size.

## Figures and Tables

**Table 1:** Baseline characteristics of the cohort, overall and by recurrence status.

Variable	All Patients (*n* = 83)	Recurrence (*n* = 50)	No Recurrence (*n* = 33)	*p*
Age, years	60 (51–66)	61 (53–68)	56 (50–64)	0.077
Body mass index, kg/m^2^	28 (26–30)	28 (27–30)	29 (26–32)	0.227
CA-125, U/mL	1157 (633–2760)	1596 (647–3755)	965 (355–2401)	0.104
Prognostic nutritional index *	40 (34–42)	38 (33–42)	41 (36–43)	0.213
FIGO stage IV	17 (20.5)	12 (24.0)	5 (15.2)	0.411
Bowel resection	20 (24.1)	10 (20.0)	10 (30.3)	0.306
HIPEC	18 (21.7)	11 (22.0)	7 (21.2)	1.000
Interval cytoreduction	9 (10.8)	2 (4.0)	7 (21.2)	0.026
Endoscopic biopsy taken	18 (21.7)	8 (16.0)	10 (30.3)	0.174
at colonoscopy	7 (8.4)	2 (4.0)	5 (15.2)	0.108
Operating time, min *	280 (220–340)	255 (215–335)	282 (229–341)	0.411
Intraoperative blood loss, mL *	600 (450–1000)	600 (400–1000)	800 (500–1000)	0.138
Length of stay, days *	8 (6–11)	9 (6–13)	7 (6–9)	0.056

Values are median (25th–75th percentile) or *n* (%). Continuous variables were compared with the Mann–Whitney U test and categorical variables with Fisher’s exact test. HIPEC: hyperthermic intraperitoneal chemotherapy. * Available in 81 patients; the prognostic nutritional index and the operative variables were missing in the same two patients. Recurrence as defined in Section 2.5 (50 events). Of the 50 recurrences, 28 were confirmed histologically and 22 were diagnosed from concordant imaging and CA-125 findings. The table is descriptive; the variables entered in the multivariable models and the reasons for not entering the others are given in the footnote to Table 5.

**Table 2:** Preoperative endoscopic assessment of the 83 patients and of the 20 who underwent bowel resection.

Finding	*n*	%
***Whole cohort (n = 83)***		
Upper gastrointestinal endoscopy performed	83	100
Colonoscopy performed	83	100
Biopsy taken at colonoscopy	7	8.4
Polyp	2	2.4
Non-specific inflammatory or reactive change	5	6.0
Biopsy taken at upper gastrointestinal endoscopy	13	15.7
Non-specific change	13	15.7
Biopsy taken at either examination	18	21.7
Biopsy taken at both examinations	2	2.4
Malignancy on any biopsy	0	0
Synchronous gastrointestinal primary identified	0	0
***Patients who underwent bowel resection (n = 20)***		
Biopsy taken at colonoscopy	4	20.0
Biopsy taken at upper gastrointestinal endoscopy	4	20.0
Biopsy taken at either examination	8	40.0
Malignancy on any biopsy	0	0

Biopsy was taken at the endoscopist’s discretion when a mucosal abnormality was seen; the endoscopic appearance itself was not recorded in structured form, so an abnormality could be identified only where it had prompted a biopsy. Because women in whom a gastrointestinal primary was found did not meet the inclusion criteria, the absence of such primaries is not a diagnostic yield.

**Table 3:** Histopathology of the 20 resected bowel specimens.

Finding	*n*	%
***Type of resection***		
Rectosigmoid resection	13	65.0
Total colectomy (one with distal ileal resection)	3	15.0
Subtotal colectomy	2	10.0
Left hemicolectomy	2	10.0
***Depth of involvement reported***		
Serosal involvement reported	15	75.0
of which mucosa explicitly described as uninvolved	10	50.0
of which submucosa also described as uninvolved	1	5.0
Tumour present, layer not specified	1	5.0
No tumour reported	4	20.0
Mucosal invasion explicitly reported	0	0 (95% CI 0–16.8)

The three depth categories are mutually exclusive and sum to 20; indented rows are subsets of the serosal category. In one of the four specimens with no reported tumour, only the mucosa and the surgical margin were explicitly described as free of tumour. Restricting the denominator to the 14 specimens in which the mucosa was explicitly described as uninvolved or no tumour was reported gives an upper bound of 23.2% for mucosal invasion. Involvement of the muscularis propria was not systematically recorded. All categories reflect what was explicitly stated in the pathology reports. CI: confidence interval (exact binomial).

**Table 4:** Type of resection, intraoperative indication and histopathology in the 20 women who underwent bowel resection.

No.	Type of Resection	Intraoperative Indication	Tumour in Specimen	Deepest Layer Reported	Mucosa
1	Rectosigmoid resection	Suspected serosal implants	No	—	Uninvolved (stated)
2	Rectosigmoid resection	Rectosigmoid involvement requiring en bloc resection	Yes	Not specified	Not described
3	Rectosigmoid resection	Suspected serosal implants	Yes	Serosa/subserosa	Not described
4	Rectosigmoid resection	Suspected serosal implants	Yes	Serosa/subserosa	Uninvolved (stated)
5	Rectosigmoid resection	Suspected serosal implants	Yes	Serosa/subserosa	Not described
6	Rectosigmoid resection	Mass narrowing the bowel lumen	No	—	Not described
7	Rectosigmoid resection	Mass narrowing the bowel lumen	No	—	Not described
8	Rectosigmoid resection	Suspected serosal implants	Yes	Serosa/subserosa	Uninvolved (stated)
9	Rectosigmoid resection	Mass infiltrating the bowel wall	Yes	Serosa/subserosa	Not described
10	Rectosigmoid resection	Rectosigmoid involvement requiring en bloc resection	Yes	Serosa/subserosa	Not described
11	Rectosigmoid resection	Suspected serosal implants	Yes	Serosa/subserosa	Uninvolved (stated)
12	Rectosigmoid resection	Suspected serosal implants	Yes	Serosa/subserosa	Uninvolved (stated)
13	Rectosigmoid resection	Rectosigmoid involvement requiring en bloc resection	Yes	Serosa/subserosa	Not described
14	Total colectomy	Suspected serosal implants	Yes	Serosa/subserosa	Uninvolved (stated)
15	Total colectomy with distal ileal resection	Suspected serosal implants	Yes	Serosa/subserosa	Uninvolved (stated)
16	Total colectomy	Mass narrowing the bowel lumen	No	—	Not described
17	Subtotal colectomy	Multisegmental involvement	Yes	Serosa/subserosa	Uninvolved (stated)
18	Subtotal colectomy	Suspected serosal implants	Yes	Serosa/subserosa	Uninvolved (stated)
19	Left hemicolectomy	Suspected serosal implants	Yes	Serosa/subserosa	Uninvolved (stated)
20	Left hemicolectomy	Suspected serosal implants	Yes	Serosa/subserosa	Uninvolved (stated)

Indications are those recorded in the operative notes, and the histopathology is that stated in the full pathology reports. No specimen showed reported mucosal invasion. Preoperative colonoscopy had been performed in all 20 women.

**Table 5:** Cox regression analysis for disease-free and overall survival.

Variable	Univariable HR (95% CI)	*p*	Multivariable HR (95% CI)	*p*
***Disease-free survival (50 events)***				
Age (per year)	1.03 (1.00–1.06)	0.039	1.03 (1.00–1.06)	0.038
CA-125 (per log_10_ unit)	1.87 (1.06–3.33)	0.032	1.69 (0.95–3.00)	0.072
Bowel resection	0.62 (0.31–1.24)	0.173	0.68 (0.34–1.38)	0.288
Stage IV	1.32 (0.69–2.53)	0.401	1.23 (0.64–2.37)	0.540
Body mass index	0.94 (0.88–1.01)	0.105	—	—
Prognostic nutritional index	0.96 (0.91–1.01)	0.107	—	—
HIPEC	0.91 (0.46–1.77)	0.773	—	—
Interval cytoreduction	0.23 (0.06–0.94)	0.041	—	—
***Overall survival (44 deaths)***				
Age (per year)	1.04 (1.01–1.08)	0.015	1.04 (1.00–1.07)	0.033
Prognostic nutritional index	0.95 (0.90–1.00)	0.052	0.95 (0.90–1.01)	0.104
Bowel resection	0.52 (0.23–1.17)	0.115	0.55 (0.24–1.24)	0.151
Stage IV	1.67 (0.84–3.32)	0.142	1.82 (0.90–3.67)	0.096
Body mass index	0.96 (0.89–1.04)	0.305	—	—
CA-125 (per log_10_ unit)	1.65 (0.88–3.12)	0.121	—	—
HIPEC	0.76 (0.35–1.64)	0.485	—	—

HR: hazard ratio; CI: confidence interval; HIPEC: hyperthermic intraperitoneal chemotherapy. The disease-free survival model comprises age, log_10_ CA-125, bowel resection and stage IV disease; the overall survival model comprises age, the prognostic nutritional index, bowel resection and stage IV disease and was fitted in the 81 patients with an available index. “—”: not entered into the multivariable model. The disease-free survival model rests on 50 events (12.5 per variable) and the overall survival model on 43 deaths in 81 patients (10.8 per variable). Each model was limited to four variables to keep about ten or more events per variable: age, bowel resection and stage IV disease were entered on clinical grounds, and log_10_ CA-125 (disease-free survival) and the prognostic nutritional index (overall survival) as the only other variables with *p* < 0.10 on univariable analysis. Body mass index and HIPEC (both models), the prognostic nutritional index in the disease-free survival model (*p* = 0.107) and CA-125 in the overall survival model (*p* = 0.121) were not entered because *p* ≥ 0.10; interval cytoreduction was not entered because its association rested on two events in nine patients.

**Table 6:** Disease-free survival with all recurrences and with histologically confirmed recurrences only.

Measure	All Recurrences (50 Events)	Histologically Confirmed (28 Events)
Median DFS, months	28	Not reached
DFS at 2, 3 and 5 years	52%, 45%, 40%	74%, 64%, 59%
Univariable HR (95% CI)		
Age (per year)	1.03 (1.00–1.06); *p* = 0.039	1.05 (1.01–1.10); *p* = 0.015
CA-125 (per log_10_ unit)	1.87 (1.06–3.33); *p* = 0.032	1.18 (0.56–2.47); *p* = 0.665
Bowel resection	0.62 (0.31–1.24); *p* = 0.173	0.69 (0.28–1.69); *p* = 0.412
Stage IV	1.32 (0.69–2.53); *p* = 0.401	0.70 (0.24–2.02); *p* = 0.510
Multivariable HR (95% CI)		
Age (per year)	1.03 (1.00–1.06); *p* = 0.038	1.05 (1.01–1.10); *p* = 0.016
CA-125 (per log_10_ unit)	1.69 (0.95–3.00); *p* = 0.072	1.21 (0.59–2.47); *p* = 0.595
Bowel resection	0.68 (0.34–1.38); *p* = 0.288	0.71 (0.29–1.78); *p* = 0.468
Stage IV	1.23 (0.64–2.37); *p* = 0.540	0.69 (0.23–2.00); *p* = 0.490
Events per variable	12.5	7.0
Recurrence by subgroup		
Endoscopic biopsy vs. none	8/18 vs. 42/65; *p* = 0.174	5/18 vs. 23/65; *p* = 0.779
Bowel resection vs. none	10/20 vs. 40/63; *p* = 0.306	6/20 vs. 22/63; *p* = 0.790

The primary analysis counts all 50 recurrences (Section 2.5); the sensitivity analysis counts only the 28 histologically confirmed recurrences, with the 22 women whose recurrence was diagnosed clinically censored at death. The multivariable models comprise age, log_10_ CA-125, bowel resection and stage IV disease. Overall survival (44 deaths) does not depend on the definition of recurrence and is shown in Table 5. Recurrence proportions were compared with Fisher’s exact test. DFS: disease-free survival; HR: hazard ratio; CI: confidence interval.

## Data Availability

The data supporting the findings of this study are available from the corresponding author on reasonable request.
